# Perioperative predictive factors of failure to rescue following highly advanced hepatobiliary-pancreatic surgery: a single-institution retrospective study

**DOI:** 10.1186/s12957-023-03257-6

**Published:** 2023-11-24

**Authors:** Masahiro Fukada, Katsutoshi Murase, Toshiya Higashi, Itaru Yasufuku, Yuta Sato, Jesse Yu Tajima, Shigeru Kiyama, Yoshihiro Tanaka, Naoki Okumura, Nobuhisa Matsuhashi

**Affiliations:** Department of Gastroenterological Surgery, Gifu University Hospital, 1-1 Yanagido, Gifu City, Gifu 501-1194 Japan

**Keywords:** Failure to rescue, Highly advanced hepatobiliary-pancreatic surgery, Perioperative predictive factors

## Abstract

**Background:**

Failure to rescue (FTR), defined as a postoperative complication leading to death, is a recently described outcome metric used to evaluate treatment quality. However, the predictive factors for FTR, particularly following highly advanced hepatobiliary-pancreatic surgery (HBPS), have not been adequately investigated. This study aimed to identify perioperative predictive factors for FTR following highly advanced HBPS.

**Methods:**

This single-institution retrospective study involved 177 patients at Gifu University Hospital, Japan, who developed severe postoperative complications (Clavien–Dindo classification grades ≥ III) between 2010 and 2022 following highly advanced HBPS. Univariate analysis was used to identify pre-, intra-, and postoperative risks of FTR.

**Results:**

Nine postoperative mortalities occurred during the study period (overall mortality rate, 1.3% [9/686]; FTR rate, 5.1% [9/177]). Univariate analysis indicated that comorbid liver disease, intraoperative blood loss, intraoperative blood transfusion, postoperative liver failure, postoperative respiratory failure, and postoperative bleeding significantly correlated with FTR.

**Conclusions:**

FTR was found to be associated with perioperative factors. Well-coordinated surgical procedures to avoid intra- and postoperative bleeding and unnecessary blood transfusions, as well as postoperative team management with attention to the occurrence of organ failure, may decrease FTR rates.

**Supplementary Information:**

The online version contains supplementary material available at 10.1186/s12957-023-03257-6.

## Background

Failure to rescue (FTR) is defined as a severe postoperative complication leading to death [1–5]. When surgery is a key component of treatment, there may be surgery-related complications that lead to FTR in some cases. In particular, highly advanced hepatobiliary-pancreatic surgery (HBPS) is more likely than general gastroenterological surgery to induce severe complications leading to FTR. The FTR rate can be used as a quality indicator of the management of postoperative complications rather than simply being indicative of complication severity. Thus, FTR is an important outcome to consider when seeking to improve treatment quality.

World Health Organization (WHO) guidelines [6] state that postoperative mortality and complication rates decreased in departments that used the WHO surgical check list [7]. In the USA, use of the National Surgical Quality Improvement Program (NSQIP) has been shown to improve surgical outcomes [8]. The Japanese Society of Hepato-Biliary-Pancreatic Surgery (JSHBPS) established systems for board certification in relation to both instructors and training institutions in Japan in 2008, which were reported to have improved highly advanced HBPS mortality rates [9].

Silber et al. suggested that both patient- and hospital-specific factors affect potential prevention of FTR [10]. However, subsequent studies have focused only on hospital-specific risk factors [11–13], whereas patient-specific predictors of FTR in highly advanced HBPS have not been adequately investigated. Recognition of factors significantly associated with FTR may improve protocols that attempt to rescue patients with severe postoperative complications. Therefore, this study aimed to identify perioperative (pre-, intra-, and postoperative) risk factors to help predict FTR following highly advanced HBPS.

## Methods

This single-center retrospective study was conducted in accordance with the World Medical Association Declaration of Helsinki and was approved by the Ethics Committee of Gifu University (approval number: 2023–018).

### Definition of FTR

The main outcome of this study was FTR, defined as in-hospital mortality after experiencing at least one severe postoperative complication. The numerator was defined as all patients who died after experiencing severe complications. The denominator included all patients who experienced severe complications. A severe postoperative complication was defined as a grade ≥ III complication according the Clavien–Dindo (CD) classification after the surgical procedure. Mortality was defined as death during hospitalization or within 90 days of the surgical procedure.

### Pre-, intra-, and postoperative variables

Pre-, intra-, and postoperative variables were included in the analysis. Preoperative variables were patient background (age, sex, body mass index); American Society of Anesthesiologists (ASA) physical status classification, active smoking, a past history of abdominal surgery, and preoperative chemotherapy; prognostic indices (prognostic nutritional index, modified Glasgow prognostic score, and systemic immune inflammation index); and patient comorbidity (Charlson risk index and type of comorbidity). Intraoperative variables were type of surgery (hepatobiliary or pancreatic surgery), operation time, blood loss, and blood transfusion. Postoperative variables were onset time of a postoperative complication, postoperative complications (pancreatic fistula, bile leakage, liver failure, respiratory failure, postoperative bleeding, and reoperation), and blood tests on postoperative day 3 (white blood cell count and C-reactive protein and albumin levels).

### Highly advanced HBPS

Highly advanced HBPSs included hepatobiliary surgeries such as hepatic trisegmentectomy, hemihepatectomy, hepatic sectionectomy (except lateral sectionectomy), hepatic segmentectomy (except S4), hepatectomy (S4a + S5 resection or hemihepatectomy) with extrahepatic bile duct resection, extrahepatic bile duct resection for congenital biliary dilatation, and hepatopancreatectomy, in addition to pancreatic surgeries such as total pancreatectomy, pancreaticoduodenectomy, distal pancreatectomy with lymph node dissection, and middle pancreatectomy.

### Statistical analysis

Continuous and categorical variables are presented as median (range) values and frequencies (percentages), respectively. Fisher’s exact test was used to compare categorical variables between two patient groups, namely, an FTR group and a non-FTR group. A Mann–Whitney *U* test was used for continuous variables. Youden’s index was used to determine the optimal cutoff value to calculate the specificities and sensitivities in receiver operating characteristic curve analysis. Variables associated with FTR following highly advanced HBPS were assessed using univariate analysis. The limit of statistical significance for all analyses was defined as a two-sided *p* value < 0.05. All statistical analyses were performed using JMP software (SAS Institute Inc., Cary, NC, USA).

## Results

This retrospective study involved 686 patients who had undergone highly advanced HBPS at the Department of Gastroenterological Surgery, Gifu University Hospital, between January 2010 and October 2022. Gifu University Hospital is a JSHBPS-certified training institution. All highly advanced HBPS surgical procedures were conducted by experienced board-certified JSHBPS-qualified surgeons.

**Fig. 1:**
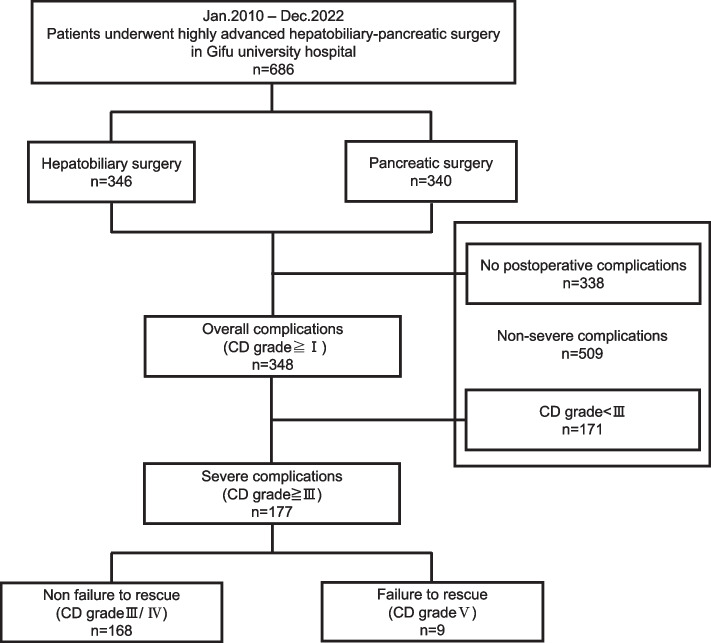
Exclusion criteria used in the study

At least one postoperative complication occurred in 348 (50.7%) patients. According to the CD grading system, 42 (6.1%) patients with grade I complications recovered without any treatment, 129 (18.8%) patients with grade II complications required antibiotic therapy, 156 (22.7%) patients with grade III complications needed radiologic intervention or re-operation, and 12 (1.7%) patients with grade IV complications and 9 (1.3%) patients with grade V complications died in the hospital. We excluded 509 patients (no postoperative complications, *n* = 338; no severe complications, *n* = 171). In total, 177 (25.8%) patients who experienced at least one postoperative severe complication, defined as grades ≥ III, were included in our study (Fig. 1).

### Surgical outcomes according to highly advanced HBPS type

Table 1 summarizes surgical outcomes according to HBPS type. The overall severe complication rate was 25.8% (177 of 686 patients), and the FTR rate was 5.1% (9 of 177 patients). The mortality rate was 1.3% (9 of 686 patients). The severe complication rate was higher in pancreatic surgery than in hepatobiliary surgery (31.8% vs. 19.9%, respectively); however, and in contrast, the FTR rates were 3.7% vs. 7.2%, respectively. Hepatic trisegmentectomy, hepatectomy with extrahepatic bile duct resection, hepatopancreatectomy, pancreaticoduodenectomy, and middle pancreatectomy showed high rates of severe complications in patients with highly advanced HBPS. FTR occurred in hemihepatectomy, hepatic sectionectomy, hepatopancreatectomy, pancreaticoduodenectomy, and distal pancreatectomy with lymph node dissection.

**Table 1:** Surgical outcomes by type of the highly advanced HBPS

	Severe complications (Clavien–Dindo classification ≧ grade III)		Failure to rescue	
	Number	Rate	Number	Rate^a^
Hepatobiliary surgeries	69	19.9%	5	7.2%
Hepatic trisegmentectomy	4	50.0%	0	0.0%
Hemihepatectomy	16	13.7%	2	12.5%
Hepatic sectionectomy	21	19.3%	2	9.5%
Hepatic segmentectomy	5	9.3%	0	0.0%
Hepatectomy with extrahepatic bile duct resection	14	42.4%	0	0.0%
Extrahepatic bile duct resection for congenital biliary dilatation	2	14.3%	0	0.0%
Hepatopancreatectomy	7	63.6%	1	14.3%
Pancreatic surgeries	108	31.8%	4	3.7%
Total pancreatectomy	4	23.5%	0	0.0%
Pancreaticoduodenectomy	86	34.3%	2	2.3%
Distal pancreatectomy with lymph node dissection	16	23.5%	2	12.5%
Middle pancreatectomy	2	50.0%	0	0.0%
Total	177	25.8%	9	5.1%

^a^Failure to rescue rate (%) = number of all patients who died after experiencing a severe complication / number of all patients who experienced severe complications

### Patient characteristics of those with severe postoperative complications

Table 2 summarizes the patient characteristics in those with severe postoperative complications. Patients in the severe complication group were significantly older, more often male, had a higher rate of pancreatic surgery, longer operation time, and more intraoperative blood loss than those in the non-severe complication group. Furthermore, the duration of hospital stay was significantly longer in the severe complication group (Supplemental Table 1).

**Table 2:** Patient characteristics of those with postoperative severe complications

	Severe complications group (*n* = 177)
Age (years)	70 (24–89)
Sex	Male: 121 (68.4%)
	Female: 56 (31.6%)
BMI (kg/m^2^)	22.0 (18.9–30.1)
ASA	1: 24 (13.6%)
	2: 133 (75.1%)
	3: 19 (10.7%)
Type of disease	Malignancy: 158 (89.3%)
	Others: 19 (10.7%)
Type of surgery	Hepatobiliary: 69 (39.0%)
	Pancreatic: 108 (61.0%)
	Open: 175 (98.9%)
	Laparoscopic: 2 (1.1%)
Operation time (min)	417 [161–949]
Blood loss (ml)	730 [55-21800]
Blood transfusion	49 (27.7%)
Pancreatic fistula	76 (42.9%)
Bile leakage	25 (14.1%)
Liver failure	6 (3.4%)
Respiratory failure	10 (5.6%)
Postoperative bleeding	25 (14.1%)
Re-operation	12 (6.8%)
Hospital stay (days)	40 (9–162)

Data are expressed as median (range) or number of patients

*BMI* body mass index, *ASA* American Society of Anesthesiologists physical status classification

### Univariate analysis to predict FTR following highly advanced HBPS

In the univariate analysis, FTR following highly advanced HBPS was significantly associated with liver-related comorbidities (*p* = 0.04), intraoperative blood loss (*p* < 0.001), intraoperative blood transfusion (*p* < 0.001), postoperative liver failure (*p* < 0.001), postoperative respiratory failure (*p* < 0.001), and postoperative bleeding (*p* = 0.02) (Table 3).

**Table 3:** Univariate analysis of prediction for FTR following highly advanced HBPS

	n	OR	95%CI	*p*-value
Age (years)				
>75	56	2.86	0.73-12.00	0.13
<75	121	1		
Sex				
Male	121	1.66	0.39-11.37	0.52
Female	56	1		
BMI (kg/m^2^)				
>24	46	0.34	0.02-1.94	0.26
<24	131	1		
ASA				
3	20	4.44	0.88-18.53	0.07
1/2	157	1		
Smoking				
Yes	99	4.18	0.72-79.29	0.12
No	78	1		
Past abdominal surgery				
Yes	76	0.65	0.13-2.55	0.55
No	101	1		
Preoperative-chemotherapy				
Yes	33	0.53	0.03-3.05	0.53
No	144	1		
PNI^a^				
>40	100	0.35	0.05-1.52	0.17
<40	77	1		
Modified GPS				
1/2	49	0.74	0.11-3.17	0.70
0	128	1		
SII^b^				
>437	88	1.26	0.32-5.27	0.73
<437	89	1		
Charlson risk index				
2+	93	1.14	0.29-4.73	0.85
0/1	84	1		
History of malignancy				
Yes	56	1.08	0.22-4.28	0.91
No	121	1		
Heart-related comorbidity				
Yes	30	1.43	0.21-6.29	0.68
No	147	1		
Respiratory-related comorbidity				
Yes	31	1.36	0.20-6.02	0.71
No	146	1		
Liver-related comorbidity				
Yes	29	4.58	1.07-18.46	0.04*
No	148	1		
Cerebrovascular-related comorbidity				
Yes	15	0.00	-2.59	0.2
No	162	1		
Diabetes mellitus				
Yes	58	1.03	0.21-4.05	0.97
No	119	1		
Chronic renal dysfunction				
Yes	12	1.78	0.09-11.09	0.62
No	165	1		
Type of surgery				
Hepatobiliary	69	2.03	0.52-8.47	0.30
Pancreas	108	1		
Operative time (min)				
>420	88	1.28	0.33-5.33	0.72
<420	89	1		
Blood loss (ml)				
>1600	25	71.06	12.02-1359.77	<0.001***
<1600	152	1		
Blood transfusion				
Yes	48	10.84	2.51-74.70	<0.01**
No	129	1		
Onset time of complication (POD)				
>12	32	3.97	0.93-15.93	0.06
<12	145	1		
Pancreatic fistula				
Yes	76	0.36	0.05-1.55	0.18
No	101	1		
Bile leakage				
Yes	25	0.00	-0.00	0.09
No	152	1		
Liver failure				
Yes	6	66.4	10.6-574.22	<0.001***
No	171	1		
Respiratory failure				
Yes	10	82	16.28-530.28	<0.001***
No	167	1		
Postoperative bleeding				
Yes	25	5.6	1.30-22.84	0.02*
No	152	1		
Re-operation				
Yes	12	1.78	0.09-11.09	0.62
No	165	1		
White blood cell on POD3 (×10^3^µl)				
>10000	73	1.84	0.47-7.66	0.38
<10000	104	1		
C-reactive protein on POD3 (mg/dl)				
>15	93	0.69	0.17-2.71	0.59
<15	84	1		
Albumin (g/dl)				
>2.8	70	1.24	0.20-4.85	0.76
<2.8	107	1		

*OR* odds ratio, *95%CI* 95% confidence interval, *BMI* body mass index, *ASA* American Society of Anesthesiologists physical status classification, *GPS* Glasgow prognostic score, *POD* postoperative day

^a^Prognostic nutritional index = 10 × albumin (g/dl) + 0.005 × the absolute lymphocyte count

^b^Systemic Inflammation Index = the absolute platelet count × the absolute neutrophil count / the absolute lymphocyte count

^*^p < 0.05

^**^p < 0.01

^***^p < 0.001

### Nine FTR cases following highly advanced HBPS

Table 4 summarizes detailed data concerning nine FTR cases following highly advanced HBP surgery. The diseases requiring surgery were hepatocellular carcinoma (HCC) in 3 (33.3%) patients; pancreatic ductal adenocarcinoma (PDAC) in 3 (33.3%) patients; and intraductal papillary neoplasm of bile duct (IPNB), metastatic pancreatic cancer from renal cancer, and cholangiocarcinoma in one (11.1%) patient each, respectively. The operating time ranged from 228 to 767 min (median, 427 min), and the amount of intraoperative blood loss ranged from 190 to 4920 ml (median, 2640 ml). A total of 7 (77.8%) patients underwent blood transfusion during the surgery. Severe postoperative complications included postoperative bleeding in 4 (44.4%) patients, liver failure in 3 (33.3%) patients, respiratory failure in 2 (22.2%) patients, and intestinal necrosis and pancreatic fistula in one (11.1%) patient each. The onset time of postoperative complications ranged from 1 to 34 days (median, 8 days). Fatal comorbidities included multiple organ failure (MOF) in 6 (66.7%) patients and hemorrhagic shock in 2 (22.2%) patients. The postoperative days to death ranged from 9 to 80 days (median, 35 days).

**Table 4:** Nine FTR cases following highly advanced HBP surgery

FTR case	Age (years)	Sex	Disease	Surgical procedure	Operation time (min)	Blood loss (ml)	Blood transfusion (ml)	Postoperative severe complications	Onset time of complication (days)	Fatal comorbidity	Postoperative days to death (days)
1	78	Male	IPNB	Right hemihepatectomy	763	2640	PRBC 280 FFP 160	Postoperative bleeding Liver failure	5	MOF	57
2	67	Male	Metastatic pancreatic cancer	Pancreaticoduodenectomy	466	4200	PRBC 1680 FFP 1920	Postoperative bleeding	8	Hemorrhagic shock	67
3	59	Male	HCC	Right hemihepatectomy	443	3315	PRBC 840 FFP 1200	Liver failure	34	MOF	68
4	77	Female	PDAC	Distal pancreatectomy with lymph node dissection	228	190	None	Respiratory failure	15	MOF	30
5	77	Female	PDAC	Pancreaticoduodenectomy	767	4920	PRBC 1120 FFP 720	Intestinal necrosis	1	MOF	80
6	79	Male	HCC	Hepatic sectionectomy	334	1630	PRBC 560 FFP 1200	Liver failure	5	MOF	35
7	67	Male	PDAC	Distal pancreatectomy with lymph node dissection	262	1840	PRBC 1120 FFP 960	Postoperative bleeding	12	Hemorrhagic shock	12
8	82	Male	HCC	Hepatic sectionectomy	407	3745	PRBC 2520	Respiratory failure	16	MOF	35
9	72	Male	Cholangiocarcinoma	Hepatopancreatectomy	427	1700	None	Postoperative bleeding Pancreatic fistula	7	Hemorrhagic shock	9

*PRBC* packed red blood cells, *FFP* fresh frozen plasma, *IPNB* intraductal papillary neoplasm of bile duct, *HCC* hepatocellular carcinoma, *PDAC* pancreatic ductal adenocarcinoma, *PD* pancreaticoduodenectomy, *MOF* multiple organ failure

## Discussion

FTR is defined as a postoperative complication leading to death [1–5]. While a higher complication rate might appear likely to lead to an increased postoperative mortality rate, Ghaferi et al. showed that differences in mortality rates were not associated with large differences in postoperative complication rates [12, 14], but with the ability of hospitals to effectively rescue patients from complications. Therefore, FTR can be considered a quality indicator of the management of postoperative complications rather than of the extent of postoperative complications alone. FTR rates have been reported to vary widely across hospitals for all procedures and are highly correlated with postoperative mortality. Hospital bed size, intensive care unit (ICU) availability, rapid response system (RRS) availability, hospital technology, nurse-to-patient ratios, average daily census, and teaching status have been found to be associated with differences in FTR rates between hospitals with very low and very high mortality rates [11–15]. These findings suggest that FTR rates might be influenced by the extent to which hospitals have well-organized multi-disciplinary teams enabling early intervention through involving endoscopists, radiologists, infection control doctors, and intensivists. Our institution, with > 600 beds and classified as a high-volume center, is a university-affiliated hospital with highly advanced technology as well as being a JSHBPS-certified training institution. Furthermore, endoscopists, radiologists, infection control doctors, and intensivists are on staff. Both ICU and RRS are available, and nursing care is provided at a ratio of 7:1. These specific characteristics of our institution are likely to have contributed to the lower FTR rates than those reported in previous studies [16–21]. Therefore, analysis of data obtained in this highly technical and well-equipped medical environment may help identify issues that need to be addressed to further reduce FTR rates following highly advanced HBPS.

Some studies have reported non-hospital-related risk factors for FTR following highly advanced HBPS. Elfrink et al. showed that factors independently associated with FTR following liver resection were age (65–80 years), an ASA physical status classification of 3, liver cirrhosis, biliary cancer, major liver resection, postoperative liver failure, cardiac complications, and thromboembolic complications [16]. Lei et al. reported that the factors predicting postoperative mortality following liver resection for hepatocellular carcinoma were the Child–Pugh score, intraoperative blood loss, and postoperative liver failure [22]. Gleeson et al. identified the following independent risk factors in FTR following PD: age, ≥ 65 years; albumin level, < 3.5 g/dl; and the development of shock, postoperative renal failure, or postoperative respiratory failure [19]. Endo et al. found that major liver resection and blood transfusion were independently associated with FTR following hepatopancreatectomy [23]. The results of this study are consistent with those of previous reports concerning perioperative predictive factors for FTR following highly advanced HBPS.

First, intraoperative blood loss of > 1600 ml and blood transfusion have been found to be predictive factors for FTR. Intraoperative blood loss is an essential consideration in surgery and has been reported to have both short- and long-term outcomes [22–25]. Nonami et al. [26] reported that blood loss was independently associated with postoperative liver failure and mortality. A strong correlation has been observed between intraoperative blood loss and blood transfusions. Yamamoto et al. [27] reported that all patients with blood loss > 1500 ml received blood transfusions in their study, including 251 liver resection cases. Therefore, considering the cutoff value for blood loss calculated in this study, it is possible to conclude that both massive blood loss and transfusion are risk factors for FTR. Homologous blood transfusion is known to increase the rate of postoperative infectious complications, owing ostensibly to immunosuppression [28, 29], with homologous blood transfusion being a significant risk factor for bacterial infection and a possible risk factor for FTR in surgically treated patients. Therefore, surgeons should maximize their efforts to decrease intraoperative blood loss and avoid unnecessary blood transfusions through the application of sophisticated surgical skills and communication with anesthesiologists.

Second, postoperative organ failure is one of the most serious postoperative complications that can lead to poor outcomes in all surgeries [30–32]. Organ failure involves organ dysfunction to such a degree that homeostasis cannot be maintained without external clinical intervention. MOF is defined as the involvement of two or more organ systems. Postoperative organ dysfunction can occur in any organ; however, the pulmonary, hepatic, cardiac, renal, and cerebral vessels are more commonly involved. In this study, we selected liver and respiratory failure as risk factors for FTR owing to their high rate of postoperative organ failure. Both types of organ failure significantly correlated with FTR in the univariate analysis. Postoperative liver failure has previously been reported to be an independent risk factor for FTR following liver resection [16–18, 22]. In addition, massive intraoperative bleeding and liver-related disease comorbidities are known causes of postoperative liver failure [16, 26], and the confounding relationship between these factors may have influenced our results.

Postoperative respiratory failure is a significant risk factor for FTR [32–34]. Postoperative respiratory failure is defined as an unplanned postoperative reintubation or prolonged postoperative intubation. Several previous studies have reported postoperative respiratory failure incidence rates ranging from 2.7 to 3.4%. Older age, ASA, pulmonary-related disease comorbidity, longer surgery, pneumonia, abdominal surgery, and diaphragmatic dysfunction have also been reported to be risk factors [32–35]. Diaphragmatic dysfunction may develop following prolonged mechanical ventilation, damage to the muscles and nerves of the diaphragm, and irritation from subdiaphragmatic abscesses or thoracoabdominal effusions. More caution may be needed to avoid respiratory failure for patients with complications that may lead to diaphragmatic dysfunction.

This study had some limitations. First, this single-center retrospective study involved a small number of FTR events, which may have resulted in selection bias and multiplicity issues in the statistical analysis. A multicenter study with a larger number of patients is required to obtain more accurate results. However, multicenter studies may show a large effect on inter-institutional disparities in terms of FTR rates. Therefore, our study concerning FTR at a single institution with a well-developed medical environment and uniform surgical indications may be of particular value. Second, this study included all patients with highly advanced HBPS and hepatobiliary and pancreatic surgeries. Each type of surgery may be associated with different risk factors for FTR. This limitation should be considered when evaluating our study results.

## Conclusions

FTR was shown to be associated with perioperative factors. Well-coordinated surgical procedures to avoid intra- and postoperative bleeding and unnecessary blood transfusions, as well as postoperative team management with attention to the occurrence of organ failure, may decrease FTR rates.

### Supplementary Information

**Additional file 1: Supplemental Table 1.** Comparison of patient characteristics between patients with and without postoperative severe complications.

## Data Availability

The datasets used in this study are available from the corresponding author upon request.

## Abbreviations

ASA: American Society of Anesthesiologists
CI: Confidence interval
FTR: Failure to rescue
HBPS: Hepatobiliary-pancreatic surgery
ICU: Intensive care unit
JSHBPS: Japanese Society of Hepato-Biliary-Pancreatic Surgery
NSQIP: The National Surgical Quality Improvement Program
OR: Odds ratio
PD: Pancreaticoduodenectomy
RSS: Rapid response system
WHO: World Health Organization
MOF: Multiple organ failure
HCC: Hepatocellular carcinoma
PDAC: Pancreatic ductal adenocarcinoma
IPNB: Intraductal papillary neoplasm of bile duct
CD: Clavien–Dindo
