# Supplementary material for: Perioperative predictive factors of failure to rescue following highly advanced hepatobiliary-pancreatic surgery: a single-institution retrospective study
Source: World J Surg Oncol. 2023 Nov 24;21:365. doi: 10.1186/s12957-023-03257-6 (PMC10668400; doi:10.1186/s12957-023-03257-6)
Supplement: Supplementary file 1 — Additional file 1: Supplemental Table 1. Comparison of patient characteristics between patients with and without postoperative severe complications. [file 12957_2023_3257_MOESM1_ESM.docx]

**Supplemental Table 1. Comparison of patient characteristics between patients with and without post-operative severe complications**

|  | Severe complications group  (n=177) | Non-severe complications group  (n=509) | *p*-value |
| --- | --- | --- | --- |
| Age (years) | 70 (24-89) | 69 (49-90) | 0.02* |
| Sex | Male: 121 (68.4%)  Female: 56 (31.6%) | Male: 298 (58.5)  Female: 211 (41.5) | 0.02* |
| BMI (kg/m^2^) | 22.0 (18.9-30.1) | 22.0 (15.1-38.8) | 0.92 |
| ASA | 1: 24 (13.6%)  2: 133 (75.1%)  3: 19 (10.7%) | 1: 64 (12.6%)  2: 396 (77.8%)  3: 48 (9.4%) | 0.80 |
| Type of disease | Malignancy: 158 (89.3%)  Others: 19 (10.7%) | Malignancy: 439 (86.3%)  Others: 70 (13.8%) | 0.36 |
| Type of surgery | Hepatobiliary: 69 (39.0%)  Pancreatic: 108 (61.0%) | Hepatobiliary: 232 (45.6%)  Pancreatic: 277 (54.4%) | < 0.01 ** |
|  | Open: 175 (98.9%)  Laparoscopic: 2 (1.1%) | Open: 491 (96.5%)  Laparoscopic: 18 (3.5%) | 0.10 |
| Operation time (min) | 417 [161-949] | 375 [126-787] | < 0.01 ** |
| Blood loss (ml) | 730 [55-21800] | 600 [0-14700] | < 0.001*** |
| Blood transfusion | Yes: 49 (27.7%)  No: 128 (72.3%) | Yes: 117 (23.0%)  No: 392 (77.0%) | 0.21 |
| Hospital stay (days) | 40 (9-162) | 15 (5-59) | < 0.001*** |

Data are expressed as median (range) or number of patients.

BMI: body mass index

ASA: American Society of Anesthesiologists physical status classification

*: *p*< 0.05 **: *p*< 0.01 ***: *p*< 0.001
